# Computational Systems Biology in Cancer: Modeling Methods and Applications

**Published:** 2007-09-17

**Authors:** Wayne Materi, David S. Wishart

**Affiliations:** 1 Departments of Biological Sciences and Computing Science, University of Alberta; 2 National Research Council, National Institute for Nanotechnology (NINT) Edmonton, Alberta, Canada

**Keywords:** cancer, computational systems biology, simulation, modeling, cellular automata

## Abstract

In recent years it has become clear that carcinogenesis is a complex process, both at the molecular and cellular levels. Understanding the origins, growth and spread of cancer, therefore requires an integrated or system-wide approach. Computational systems biology is an emerging sub-discipline in systems biology that utilizes the wealth of data from genomic, proteomic and metabolomic studies to build computer simulations of intra and intercellular processes. Several useful descriptive and predictive models of the origin, growth and spread of cancers have been developed in an effort to better understand the disease and potential therapeutic approaches. In this review we describe and assess the practical and theoretical underpinnings of commonly-used modeling approaches, including ordinary and partial differential equations, petri nets, cellular automata, agent based models and hybrid systems. A number of computer-based formalisms have been implemented to improve the accessibility of the various approaches to researchers whose primary interest lies outside of model development. We discuss several of these and describe how they have led to novel insights into tumor genesis, growth, apoptosis, vascularization and therapy.

## Background

Living organisms are complex systems. Nowhere is this complexity more evident than in the genesis and development of cancer. While cancer may originate from genetic and molecular changes that occur in a single cell, the subsequent proliferation, migration and interaction with other cells is crucial to its further development. In their landmark paper, Hanahan and Weinberg described six hallmarks they thought necessary for the transition from normal cells to invasive cancers (Hanahan and Weinberg, 2000). These included: 1) self-sufficiency in growth signals, 2) insensitivity to growth inhibitory signals, 3) evading apoptosis, 4) limitless replicative potential, 5) sustained angiogenesis, and 6) tissue invasion and metastasis. While genetic instability was not explicitly included in this list, it was included as an implicit enabling alteration that might start a normal cell down a mutagenic pathway leading to the acquisition of one or more of these essential characteristics.

The molecules that govern the cell growth and division cycle in response to external and internal signals are numerous and interact through complex, multiply-connected pathways over a wide range of temporal and spatial scales. Tumors reflect this complexity in that they are composed of several different cell types that interact to create malignant growth (Burkert et al. 2006). Despite the widespread acceptance of this complexity, the majority of biological and biomedical studies still utilize a strictly reductionist approach, focusing on the interactions of at most a few genes or proteins in each experiment. Systems biology, an integrative discipline that attempts to describe and understand biology as systems of interconnected components, has arisen partly as a response to these traditional reductionist approaches. Systems biology is a young field made possible by the explosion of data from genomic, transcriptomic, proteomic and metabolomic techniques developed within the last decade (Hollywood et al. 2006; Bugrim et al. 2004; Jares, 2006).

Computational systems biology, which is a sub-discipline of systems biology, has developed both as a tool supporting the processing of these massive amounts of data and as a modeling discipline, building upon this “omic” data in order to predict biological behavior (Ideker et al. 2001a; Alberghina et al. 2004). Not surprisingly, both experimental and computational systems biology approaches have provided fruitful insights into cancer.

This review provides an overview of how computational systems biology can be, and is being used to model cancer at multiple levels and scales, ranging from molecules to cells to tissues. Specifically, we begin with a general description of the different computational modeling methods that can be used along with a discussion of their relative strengths and weaknesses. Following that, we describe some of the emerging formalisms, software and standards for representing biological systems. Finally, we provide a number of examples illustrating how computational systems biology has enriched our understanding of a variety of cancer-related processes including genetic instability, tumor growth, apoptosis, angiogenesis and anti-cancer therapy. Overall it is our hope that this review will provide an improved understanding of modeling issues and thereby assist the reader in selecting an appropriate method for their own research.

## Approaches to Computational Systems Modeling

To be truly useful to a biologist or physician, computational modeling should: 1) produce useful predictions or extrapolations that match experimental results; 2) permit data to be generated that is beyond present-day experimental capabilities; 3) allow experiments to be performed *in silico* to save time or cost; 4) yield non-intuitive insights into how a system or process works; 5) identify missing components, processes or functions in a system; 6) allow complex processes to be better understood or visualized and 7) facilitate the consolidation of quantitative data about a given system or process.

**Figure 1:**
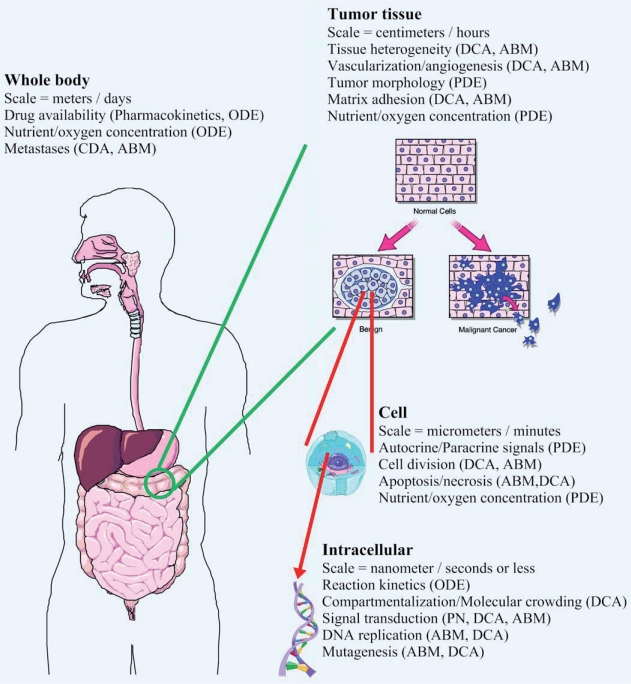
Issues of scale in modeling cancer. From whole organism to tumor tissue to individual cells to the molecules of replication and metabolism, modeling tumors spans about nine orders of spatio-temporal magnitude. Shown above are some of the modeling issues which need to be addressed at each level of simulation. Each text box includes the relevant spatio-temporal scale and modeling issues encountered at that level. Appropriate modeling approaches to address each issue are shown in brackets. Building hierarchical systems of inter-related models is still a primary challenge to modern researchers. ODE – Ordinary differential equation system, PDE – Partial differential equation system, DCA – Dynamic cellular automaton, PN – Petri net system, ABM – Agent based model.

Simulations encompass many different spatial and temporal scales, ranging from nanometers to meters and nanoseconds to days (Fig. 1). Processes that occur over very small dimensions (nm) or short time periods (ms) are often referred to as “fine grain” models, while processes occuring over longer time periods (s) or larger (mm or cm) dimensions are called “coarse grain” models. A fundamental challenge to computational systems biology is to develop models and modeling tools that can deal with this wide range of granularity. In this review we will describe some of the newer or more innovative modeling techniques that are being developed to permit both temporal and spatio-temporal modeling over this wide range of scales, including: 1) systems of ordinary differential equations (ODEs), partial differential equations (PDEs) and related techniques, 2) Petri nets, 3) cellular automata (CA), dynamic cellular automata (DCA) and agent-based models (ABMs) and 4) hybrid approaches. Figure 1 presents an overview of scaling issues in modeling cancer and indicates which approaches are particularly well-suited to dealing with each area.

Building models of complex biological processes is an iterative process that requires considerable attention to detail. The network topology or structure of a model may arise through literature surveys or directly by computational analysis of high-throughput data (Wang et al. 2007—[Epub ahead of print]). In many instances such analyses may reveal novel regulatory or signal transduction interactions whose kinetics and stoichiometry is unknown (Janes et al. 2005; Kumar et al. 2007). Quantitatively accurate modeling requires explicit values for many variables including molecular concentrations, cellular distribution of molecules, reactions rates, diffusion rates, transport rates and degradation rates. While many of these can be estimated from the literature or various online databases, a number of parameters often remain unknown at the start of any simulation. As a result, many modeling processes require that one provide estimates for key parameters. Usually “best guess” first order estimates can be used and then fine-tuned using a well-understood instance of the model as a comparison. Parameters are iteratively adjusted on subsequent simulations until the model accurately reflects the known test case (Ideker et al. 2001a; Kunkel et al. 2004; Ideker et al. 2006). This period of validation is always required where any unknown parameters exist. However, a detailed discussion of network discovery and the model refinement/validation process is beyond the scope of this review.

### Computational modeling using differential equations

Biological systems are essentially multicomponent chemical reactors and thus can be represented as systems of chemical reactions. This view permits mathematical analysis using powerful techniques developed from chemistry. Many standard biochemistry texts provide thorough derivations of ordinary differential equations (ODEs) for both simple and complex reactions. In fact, ODE based modeling is the most common simulation approach in computational systems biology, reflecting both its rigor and adaptability (Kitano, 2002; De Jong, 2002).

Simple ODEs may have exact solutions. However, most complex ODEs do not have exact solutions and must be solved numerically. Based on methods first derived by Newton and Gauss, numerical integrators utilize linear approximations of smooth curves over small time intervals to compute subsequent values of reactant concentrations. Improving the accuracy of these linear estimates may require using smaller time intervals, leading to computationally intense processes that use considerable machine time. Various methods have been derived to improve the speed and computational accuracy of these approximate methods, including Runge-Kutta algorithms and implicit methods (for so-called “stiff” differential equations). These algorithms are encapsulated in such publicly-available packages as the LSODA (Liver-more Solver for ODEs with Automatic method switching for stiff and non-stiff problems) or CVODE (C Variable-coefficient ODE solver) integrators (http://www.llnl.gov/CASC/odepack/).

In addition to improved methods for solving general systems of ODEs, models of biological systems can take advantage of features specific to such systems. One such class of ODEs is called delay differential equations (DDEs). DDEs can reduce the computational effort required to model signal cascades, such as phosphorylation-dephosphorylation cycle networks (Srividhya et al. 2007). This allows the modeler to replace an intermediate signaling molecule (and all the ODEs associated with it) with a time delay term in the ODEs immediately preceding or following in the pathway. As long as the concentration of the intermediate is not a molecule whose concentration is tracked within the system, this results in no loss of information. By removing terms representing intermediate components in multi-stage processes, such as signal transduction cascades or mitosis, this approach reduces the number of equations to be solved and leads to more efficient processing.

Systems of ODEs have been used to mathematically model a wide variety of processes including metabolic pathways (Ideker et al. 2001b), mitosis in yeast (Tyson, 1991) and genetic regulatory circuits (Elowitz and Leibler, 2000). For example, as a representative MAP kinase signal transduction cascade, the ERK/MAPK pathway has been modeled independently by several researchers using ODE systems (Orton et al. 2005). Surprisingly, differences in construction details of various models, including number of molecular species and reactions, still led to similar results and predictions. It has been suggested that this is likely an indication of the general robustness of the ODE approach (Orton et al. 2005). Alternatively, this may reflect the inherent robustness of biological systems themselves.

In many cases of biological or chemical modeling the kinetics can be described by a series of logarithmic or power law functions. As a result power law approximations of ODE systems have been developed to improve the computational processing of this very common class of kinetic models. In this approach the rate of a reaction in the steady state can be estimated using Taylor series approximations of ODEs (Savageau, 1991). This leads to a system of non-linear equations where each rate is a product of non-integer powers of reactant concentrations and a coefficient. In a logarithmic coordinate system where the slope of the line represents the kinetic order of a particular reaction, the non-linear equations can be readily transformed into a system of linear equations which can be numerically solved with little computer effort. These so-called S-systems have been used to model the TCA cycle in *Dictyostelium discoideum* (Shiraishi and Savageau, 1992; Shiraishi and Savageau, 1993) and to compute strategies for improving L-carnitine production in *E. coli* by altering media and bioreactor conditions (Sevilla et al. 2005).

S-system models can also be used to estimate coefficients and exponents associated with individual components when reaction rates are unknown. One such model used simulated annealing (SA), a process whereby initial estimates of coefficients and exponents are stochastically perturbed while the system is slowly “cooled” from an initial high pseudo-temperature (Gonzalez et al. 2006). SA methods permit the system to explore beyond local minima while the pseudo-temperature is high, then to converge towards local minima upon cooling. During the cooling period, model outcomes are compared to actual biochemical profiles using a least-squares error analysis. If the error for the current simulation is equal to or lower than that for the previous simulation, the current simulation is automatically accepted. If the new error is greater, it may still be accepted with a probability that is proportional to the pseudo-temperature.

### Issues in ODE-based models

Both conventional systems of ODEs and their Power Law approximations do not automatically reflect compartmentalization, transport and diffusion of molecular species unless explicitly specified. This is because reaction kinetics equations, upon which these mathematical models are formulated, assume steady-states in well-mixed solutions with abundant reactants and few enzymes. The simplifications in standard representations of enzymatic reactions (such as Michaelis-Menton equations) incorporate assumptions about the relative rates of intermediate steps which are independent of transient localized differences in concentrations. In reality, the cellular space even inside bacteria is crowded with macromolecules, having 300 to 400 g/l of macromolecules (e.g. protein and RNA) occupying 20 to 30 percent of cytoplasmic space, compared to the 1 to 10 g/l under which biochemists normally study reaction kinetics (Ellis, 2001). Macromolecular crowding strongly affects assumptions about diffusion implicit in ODE-based modeling systems. For instance, one basic assumption in most models is that local concentrations of reactants or catalytic enzymes within the compartment are homogeneous. Even if local concentrations are explicitly modeled, diffusion rates for species of considerably different physical size are often assumed to be identical. Clearly, under crowded conditions, small molecule diffusion will be less impeded than that of larger proteins and protein complexes (Ellis, 2001).

Macromolecular crowding also affects reaction kinetics. Equilibrium rate constants for macromolecular association reactions under crowded conditions can increase by two to three orders of magnitude compared to dilute concentrations (Ellis, 2001). Thus the net effect of crowding on enzyme-catalyzed small-molecule reactions is a complicated function of the reduction in the rates of reactant diffusion and the promotion of enzyme-reactant transition complexes and is different for each reaction.

Within the cell, macromolecular crowding also leads to the formation of numerous non membrane-bound three-dimensional compartments and one-dimensional channels where reactions may occur. In addition, membranes can act as two-dimensional surfaces, leading to localized concentrations of proteins and complexes (Clegg, 1984; Srere et al. 1989). While conventional ODE systems and Power Law approximations do not inherently incorporate macromolecular crowding, they can be readily adjusted to a more accurate form using so-called “modified fractal-like kinetics” (Schnell and Turner, 2004; Kopelman, 1988). This adjustment introduces a time-independent rate constant that reflects the dimensionality of the reaction chamber (a measure of macromolecular overcrowding and compartmentalization).

Many biological molecules are present in very low concentrations (1 – 10 nM within the cytoplasm, which translates to fewer than 100 molecules per cell). Classical ODE solvers are highly unstable and may fail to accurately reflect the granularity and discrete system behaviour typically found at such low concentrations. Because of this, some modelers have added “noise terms” to their systems of equations by using so-called stochastic differential equations (SDEs) (Meinhardt and De Boer, 2001; Chen et al. 2005). An alternative and widely-used approach to introducing stochasticity into a system of ODEs is to introduce a “master equation” derived from a “grand probability function” (Gillespie, 1976). The Stochastic Simulation Algorithm (SSA) is a simple method for selecting which reactions will occur in a given time interval based on such a master equation. For each reaction, the SSA method calculates *P*(*t,*μ), the reaction probability density function; where *P*(*t,*μ)*dt* = the probability that, given the state (X_1_, X_2_, … X_n_) at time t, the next reaction, will occur in the infinitesimal time interval (t + τ, t + τ + dτ). For any reactant in any time interval in a chemical reaction system *P*(τ*,*μ) can be calculated in a straightforward fashion from a uniform pseudorandom generator (see Gillespie (1976) for a complete derivation).

SSA methods have been used to model a variety of processes, including PKC signal transduction (Manninen et al. 2006), MAPK signal transduction (Chatterjee et al. 2005), and *Hox* gene expression in the developing vertebrate hindbrain (Kastner et al. 2002). Although SSA methods are accurate, they are computationally intensive. Chatterjee et al. have developed an explicit binomial tau-leap method to accelerate SSA models by two to three orders of magnitude and demonstrated this improvement in a MAPK cascade simulation (Chatterjee et al. 2005). This algorithm computes transition probabilities per unit time for a reaction system, then allows a “bundle” of events sampled from a binomial distribution to occur simultaneously in the next time interval τ. This bypasses the dominating effects of fast kinetics reactions in the SSA model and emphasizes slower reactions that are likely of more interest to the modeler. Gillespie has recently reviewed several improvements to the original SSA method as well as implicit tau-leap methods (Gillespie, 2007). He has also introduced slow-scale SSA methods, an approximation that is applicable only for stiff ODE systems but which also result in accelerations of two to three orders of magnitude in solution time.

A key limitation of ODE-based models is that they only allow for one independent variable in a system. As a result, most ODEs represent changes in the concentration of some chemical species varying over the independent variable time. Systems of partial differential equations (PDEs) must be used if one wishes to incorporate explicit spatial distribution of components into a model. PDEs have been used to model several processes in cancers, including chemotactically-directed tumor growth (Castro et al. 2005), growth factor-stimulated glioblastoma growth (Khain and Sander, 2006), the tumor-immune system interaction (Matzavinos et al. 2004), and tumor growth along tubular structures (Marciniak-Czochra and Kimmel, 2007). Due to the increased complexity and number of variables, PDE solvers are even more computationally intensive than ODE solvers.

Historically, the effort and mathematical skill required to set up a useful ODE or PDE model put this approach beyond the reach of most experimental biologists. However, recent advances in graphical interface (GUI) design, improved standards in displaying and generating reaction models along with the development of standardized mark-up languages such as SBML (Systems Biology Mark-up Language) and Cell-ML (Cell Mark-up Language) are making the generation and exchange of interesting biological models relatively simple (Schilstra et al. 2006). A large repository of ODE-based Cell-ML metabolic and cell signaling models has been compiled at the Cell-ML model repository website (http://www.cellml.org/examples/repository/) as well as in the JWS online system (http://jjj.biochem.sun.ac.za) which is part of the silicon cell project (http://www.siliconcell.net) (Snoep et al. 2006). Additionally a large number of SBML models are located at the BioModels website (http://www.ebi.ac.uk/biomodels/). Table 1 provides a list of common ODE simulation packages, many of which are compatible with SBML or Cell-ML.

### Computational modeling using petri nets

Petri nets are a discrete alternative for representing time-dependent processes such as those occuring within biological systems (Moore et al. 2005). Petri nets, which were originally developed in the 1960’s, have long been used to model discrete distributed flow systems such as data-communications networks and manufacturing processes. It wasn’t until 1993 that biologists realized that this modeling approach could be easily adapted to representing biological systems (Reddy et al. 1993). A Petri net contains two kinds of nodes, called “places” and “transitions”, represented graphically by circles and rectangles, respectively. In a molecular model each place is a species of molecule with some number of tokens inside, representing the number of molecules or concentration of that species (called the “marking” of that place). Transitions represent reactions. Places are connected to transitions by arrows (or “directed arcs”) either from source (input) places into the transition or from the transition to product (output) places. The stoichiometry of a reaction is indicated by a weight on the arc. Because it is a discrete system, it is driven in stepwise fashion by implicit time increments. A transition “fires” (i.e. the reaction occurs) when the markings at all its input places are greater than the weights on its input arcs (ie when there are enough source molecules), producing product of the appropriate weights on its output arcs (which are subsequently stored in the product places).

Petri nets were originally designed to model discrete processes but later enhancements have added the ability to deal with continuous quantities (Goss and Peccoud, 1998; Matsuno et al. 2003). In addition, the basic Petri net formalism has been extended to deal with many of the complex issues that also arise in ODE-based models (Pinney et al. 2003). Hybrid Petri Net and Functional Hybrid Petri Net (FHPN) models allow markings in places to take either discrete or continuous values, thus permitting equivalent modeling power to ODE-based systems (Doi et al. 2004; Matsuno et al. 2003). Timed Petri Nets allow the implicit incorporation of deterministic delays in firing transitions, similar to those incorporated in DDE systems. Stochastic Petri Nets control transition firing with an exponentially-distributed time delay, equivalent to “chemical master equation” approximations of stochastic behavior in ODEs (Goss and Peccoud, 1998; Kurtz, 1972). Colored Petri Nets, which are an extension of Hybrid Petri Nets, allow for the definition of mathematical relationships inside transitions governing the rate of firing (Lee et al. 2006). Finally, Hierarchical Petri Nets are intended to support the composition of more complex models using combinations of previous models. No single implementation provides support for all variations. Further, compartments can only be represented explicitly, where different places represent the same chemical species in different compartments.

Petri Nets have been used to model a wide range of biological processes, including qualitative modeling of apoptosis (Heiner et al. 2004), iron homeostasis (Sackmann et al. 2006) and the yeast mating response (Sackmann et al. 2006). FHPNs have been used to model several biochemical processes, including the *E. coli lac* operon (Doi et al. 2004), urea cycle disorders (Chen and Hofestadt, 2006) and p53 transcriptional activity (Doi et al. 2006). Koh et al. have used FHPNs to model the AKT and MAPK pathways (Koh et al. 2006). Using an evolutionary parameter selection technique and pathway decomposition these authors were able to determine optimal parameters for the model. Subsequent simulations suggested that Akt-MAPK crosstalk is required for enabling the MAPK pathway. Colored Petri Nets have recently been used for quantitative modeling of the EGF signal transduction pathway in an efficient and dynamic manner using rate equations inside transitions that are highly reminiscent of ODE-based SBML or CellML systems (Lee et al. 2006).

### Computational modeling using cellular automata and agent based models

An alternative approach to modeling the complex systems of discrete molecules that are found in living organisms is to use cellular automata (CA) to represent individual molecules and the rules that govern their interactions. Cellular automata (CA) are simple computer simulation tools that can be used to model both temporal and spatio-temporal processes using discrete time and/or spatial steps. Cellular automata were invented in the late 1940’s by von Neumann and Ulam (Rucker and Walker, 1997) who conceived of an infinite lattice of points (or cells), each capable of a finite number of states. Each cell is connected to a finite number of neighbors whose collective states at time *t_n_* induce it to assume a new state at time *t_n+1_* in a specified manner. In biological systems the lattice represents two- or three-dimensional volumes in space and each cell can contain one molecule or biological cell (or sometimes more). So-called “lattice-free” systems use the lattice to represent real, physical space and individual entities may span more than one cell (i.e. more than a single x,y,z coordinate). Time is a discrete entity in CA models. At time *t_n_* the state and neighbors of each cell are tallied and rules applied to determine the state transition for that cell in the next interval *t_n+_*_1_.

Rules of varying complexity govern the interactions between adjacent or nearby molecules. Rules may be quite simple, for example, specifying binding of adjacent molecules with a certain probability (Wishart et al. 2005). Alternatively, more complex rules can be formulated. For example, interactions of molecules may take place in a distance-dependent manner, representing biophysical relationships more accurately (Broderick et al. 2005).

In order to realistically simulate complex systems of reacting molecules, stochasticity needs to be added to the deterministic rules of the original cellular automata formalism. Dynamic Cellular Automata (DCA) permit “Brownian-like” motion of individual molecules through the incorporation of a random number generator which selects a direction of motion in the next time step (Wishart et al. 2005). Depending on the implementation of the DCA algorithm, molecules may move one or more cells in a single time step.

Chemical reaction rates are emergent properties of DCA models although molecular reaction probabilities may be derived from conventional reaction rates. However, given the problems in deriving biologically relevant reaction rates that take into account macromolecular crowding, extremely low concentrations and compartmentalization, it may be more accurate to compute parameters that match the biochemical profile of a well-known test model.

DCA models have been used to model a wide variety of processes including diffusion (Kier et al. 1997; Wishart et al. 2005), micelle formation (Kier et al. 1996b), basic enzyme kinetics (Kier et al. 1996a; Wishart et al. 2005), myxobacteria aggregation (Sozinova et al. 2005), osmotic shock (Broderick et al. 2005) and HIV/AIDS progression and treatment in single patients (Sloot et al. 2005). Both metabolic processes and simple genetic circuits have been modeled using estimates for reaction probabilities based on general kinetics or empirical values (Wishart et al. 2005). The results in all these cases have proven to be surprisingly accurate despite the apparent simplicity of the models, proving the power of the DCA approach.

Models of complete cells, even simple bacteria, could potentially incorporate billions of individual molecules. Interactions between molecules must be specified in a computationally efficient manner if such models are to compete with solution times for the mere hundreds of equations potentially found in comparable ODE systems. Nonetheless interactions must be firmly based on real forces or accepted approximations. For example, a recent DCA model of lipid bilayer dynamics in a 60 nm diameter proto-cell with over 10,000 individual components used the Lennard-Jones potential as the basis for attractive and repulsive forces to realistically model the membrane (Broderick et al. 2005).

Agent Based Modeling (ABM) is similar in concept and design to Dynamic Cellular Automata. In ABMs genes, proteins, metabolites or cells can all be “agents”. Agents are allowed to interact with each other over space and time according to a pre-defined set of rules. The motions may be directed or random (Brownian) and the rules may be simple or highly complex. Unlike CA models, agent based systems do not formally require spatial grids or synchronized time steps, although practical coding considerations usually force these constraints on ABMs. Space is usually represented in a lattice-free grid. ABMs share many of the same advantages and disadvantages as DCA or CA models. Agent-based models have been used to simulate bacterial chemotaxis (Emonet et al. 2005), to model the calcium dependent cell migration events in wound healing (Walker et al. 2004) and to predict clinical trial outcomes of different anti-cytokine treatments for sepsis (An, 2004).

## Hybrid Approaches

ODE, Petri Net, DCA and ABM approaches all have their unique advantages and disadvantages. While DCA and ABM systems have molecular-grain accuracy and implicit simulation of compartmentalization, diffusion and stochasticity, they can also be computationally more intensive as the number of components in a model can increase rapidly (Ridgway et al. 2006). ODE systems ideally represent dilute reactants in single compartments, though efficient methods have been developed for approximating more biologically realistic models. Nevertheless, ODE systems do not generally capture the true granularity or stochasticity of living systems and do not usually provide adequate visual feedback so important to developing clear conceptualization (Wishart et al. 2005).

A number of researchers have recommended hybrid or hierarchical hybrid systems to combine the strengths of both discrete and continuous approaches (Coveney and Fowler, 2005; Ridgway et al. 2006; Sorger, 2005). For example a simulation of bacterial biofilm development was developed where soluble substrates were represented by PDE systems in four spatio-temporal dimensions while bacterial cell growth was modeled seperately with a deterministic CA (Picioreanu et al. 1998). This model accurately reflected global oxygen consumption as well as the concentration profiles of substrate and biomass. The distribution of the bacterial cells in relation to substrates and inoculation density were also accurately predicted. A primary benefit from hybrid models is the ability to integrate processes that occur rapidly (e.g. diffusion) with processes that can take days (e.g. cell growth and migration). This mixing of scales of time and space is normally one of the most significant impediments to accurately modeling biological processes using reasonable computer power (Coveney and Fowler, 2005).

Biological systems can also be viewed as complex control systems whose rules can be represented using so-called “fuzzy logic” (Sproule et al. 2002). Fuzzy logic permits the use of qualitative terms such as “high” or “low” concentration to be incorporated into models. A hybrid model incorporating inference rules and fuzzy logic has recently demonstrated the utility of this approach by modeling *sonic hedgehog* signaling in the development of medulloblastomas (Bosl, 2007).

## Formalisms for Representing Biological Systems

Most biologists are not comfortable representing molecular systems using many of the mathematical tools described above. Further, most models have been developed without consideration for integration with models developed by other groups. To address these issues, Hucka, Finney and others formed a *Software Platforms for Systems Biology* forum in 2000 where they first proposed the development of SBML or Systems Biology Markup Language (Hucka et al. 2003; Finney and Hucka, 2003). SBML is a simple formalism (language) for describing networks of chemical reactions occurring inside biological entities. It uses the widely-accepted XML (eXtensible Markup Language) representation to define compartments, molecular species, reactions, parameters and rules (Webb and White, 2005).

Representing a simple reaction such as $A+B\overset{k_{1}}{\to }C$ is not a trivial process in SBML. Even ignoring supporting statements, describing this single chemical equation still requires a relatively large amount of SBML code. In addition to being long, the code is largely unreadable to the novice. Level 2 enhancements to the SBML definition (specification available at http://source-forge.net/project/showfiles.php?group_id=71971) which incorporate Content MathML (http://www.w3.org/TR/MathML2/) and Resource Description Framework (RDF – http://www.w3.org/RDF/) for metadata description, only made this situation worse. Fortunately a large number of support programs have enabled biologists to define components of an SBML system using simple graphical descriptions of reactions and compartments that are much more easily interpretable. Links to web-sites supporting SBML models can be found at http://www.sbml.org.

For simulation purposes SBML is strictly ODE-based. Despite this restriction, a large variety of SBML-related modeling programs have arisen for the purpose of actually running simulations defined in the formalism. SBML handles compartmentalization of reactants through its use of a *KineticLaw* reaction definition which incorporates compartment volumes into standard reaction rate equations (Finney et al. 2006). However, while rate equations for simple compartmentalized reactions may be converted easily to a corresponding *KineticLaw,* more complex reactions may require specific knowledge of compartment transfer rates. Both continuous and discrete models can be defined in SBML though they require different representations of the corresponding rate equations. A repository of published and unpublished SBML models is maintained at http://www.ebi.ac.uk/biomodels/ which can also be reached from the Biomodels web site http://www.biomodels.net/ (Le Novere et al. 2006).

CellML is a an example of another XML-based formalism developed through the International Union of Physiological Sciences (IUPS) Human Physiome Project (Hunter et al. 2002; Lloyd et al. 2004). CellML models networks of interconnected components whose behavior is described by mathematical equations written in Content MathML 2.0, a subset of which is embedded within the CellML framework. These features make CellML particularly amenable to modeling electrophysiological systems though it readily incorporates chemical reactions and gene networks. In addition, CellML provides for the inclusion of metadata (i.e. data describing the model, itself) within the model. This may include data derived from publications and ontological or semantic descriptions, such as keywords. Many of these features have now been incorporated into SBML Level 2 as well. An important part of the CellML philosophy (besides providing a clear, consistent, verifiable formalism) is to encourage hierarchical composition of models. This allows one to combine previously developed models in workable combinations (Lloyd et al. 2004). CellML’s encapsulation grouping structure is somewhat reminiscent of modern object-oriented programming languages. Version 1.1 of CellML also introduced the *import* feature to allow for components and connections to be reused in new models.

The modeling power of CellML is based on the ability to explicitly define the mathematical relations between all components. Compartments within a cell are considered components into which other components may be grouped. Thus, movement of components between compartments must be defined explicitly. Stochastic modeling is not yet supported by CellML nor is the modeling of discrete objects. The CellML and SBML project teams are cooperating to incorporate each other’s strengths, where possible, and to provide for models in one formalism to be translated into the other (Schilstra et al. 2006). Complete specification of the CellML language can be found at http://www.cellml.org/specifications and over 350 models are currently contained in a repository at http://www.cellml.org/models/.

No single formalism represents the Cellular Automata approach to modeling Usually the definition of each CA formalism is encapsulated in its specific implementation. Recently, however, some effort has been made to formalize a more consistent approach to generating and sharing hybrid CA models of cellular processes (Cho et al. 2005). This hybrid approach allows both discrete and continuous components, constraints, stochasticity, regulation of transcription and mutations in a mathematical framework. However this system has only been used to model a small reaction system and it is uncertain whether it could be readily extended to more complex processes. Overall the CA field continues to be dominated by insular approaches to specific problems. This should not be too surprising given the fact that the cellular automata approach is more concerned with an implementation philosophy rather than notational representation. On the other hand proponents of CA emphasize that continuous values are actually emergent properties of discrete processes and that a CA approach would result in truer models (Wolfram, 2002). Others have suggested that multiple formalisms incorporating, for example, ODEs for physiological and chemical changes but CA formalisms for discrete cells in a tissue may lead to the best compromise between biologic accuracy and implementation speed (Defontaine et al. 2004).

Frequently, translation into a specific formalism is the most difficult step in converting a drawing of some process into a formal model for that process. The notation systems for all formalisms look nothing like the diagrams that biologists and biochemists are used to (with the exception that biochemists may be somewhat familiar with ODE representations of chemical reactions). Fortunately computer-based graphical tools have been developed permitting the entry of a model with symbols familiar to biologists (or easily learned) and subsequent machine conversion into a particular formalism. A number of these are listed in Table 1 along with web sites from which they can be downloaded or run.

In addition to defining a model, simulation requires some computer program to run the model over a period of time or iterations producing output for interpretation. Output usually consists of graphs of the concentration of some component over time (or number of molecules, for discrete models) but may also include dynamic representations of the system or other analysis. The programs listed in Table 1 all include or can be linked to a variety of simulation modules. Reviews comparing specific features of some of these systems have been published (Peleg et al. 2005; Alves et al. 2006). In addition, a number of specific SBML tools have been developed, including SBML ODE Solver (Machne et al. 2006), MathSBML (Shapiro et al. 2004), SBML ToolBox (Keating et al. 2006), SBW-MATLAB interface (Wellock et al. 2005), SBML-PET (parameter estimation tool) (Zi and Klipp, 2006), and various other tools for programmers wishing to connect to SBML or the Systems Biology Workshop (SBW) (Gillespie et al. 2006). The availability of these tools (http://sbml.org/index.psp) should make computational systems biology far more accessible to a much larger community of life sciences researchers.

## How has Modeling Informed us About Cancer?

As was noted earlier biological simulations should not be considered as simple academic exercises. Rather they should aim to inform researchers about some unobvious characteristic of the simulated process. It is appropriate to ask, then, how models of cancer have led to novel insights into the disease. In the following section we review how modeling and computational systems biology has contributed to our understanding of the underlying molecular, cellular and tissue-level mechanisms of cancer.

### Genetic Instability

The contribution of genetic instability to the development of cancer is controversial. Estimates of the number of mutations acquired in cancer cells range from 10^4^ at the lower limit to a maximum of about 10^12^ (Abbott et al. 2006). A simple ODE model has been developed to explore the kinetics of tumor progression and to compare the importance of genetic instability compared to other factors such as avoidance of apoptosis, increased growth rate or angiogenic signaling (Spencer et al. 2004). The 17 equations in this model incorporated a mutagenesis rate which resulted in various advantageous mutations. Following acquisition of the genetic instability mutation, the global rate of mutagenesis was increased. Surprisingly, the model demonstrated that genetic instability was only important in the development of late-stage sporadic tumors, as it confered no general survival advantage to altered cells in the early stages of cancer.

A separate study led to a similar conclusion on the basis of examination of the karyotype of populations of cancer cells (Heng et al. 2006). Rather than instability associated with individual genes, large-scale chromosomal instability was shown to make a more important contribution to early development of pre-cancerous cells. Clonal expansion and avoidance of apoptosis was critically required for early tumor development as these characteristic allowed unstable cells to exhibit any acquired survival advantages. These ODE models did not, however, incorporate removing replication limits on cancerous cells nor metastasis, but proposed that agent-based approaches might model these characteristics better.

A direct test of increased genetic instability in tumor tissues has recently been conducted in transgenic mice that produce mammary tumors with high frequency (Stringer et al. 2005). These mice were also transgenic for a mutant allele of human placental alkaline phosphatase incorporating an insertion of an 11 base pair G:C tract. This insertion caused a frameshift which rendered the protein inactive. Loss or insertion of base pairs due to an increased mutation rate (resulting from genetic instability associated with cancerous cells) could restore gene function and allow such cells to be visualized *in situ* in tissue sections. A simple probablistic computer model predicted that many islands of individual staining cells, rather than only a few large clusters of staining cells, should predominate in tumors if genetic instability were more important in early-stage cancers than increased proliferation or apoptotic avoidance (Stringer et al. 2005). In the aggregate, across 17 tumors examined, the frequency of cell clusters of the predicted size matched the predictions of this simple genetic model. However, individual tumors exhibited extreme variability in cluster population, suggesting that hyperproliferation and survival were more important than genetic instability in many tumors.

A more recent stochastic cellular automata model based on Hanahan and Weinberg’s “hallmarks of cancer” was proposed to resolve the issue of the importance of genetic instability (Spencer et al. 2006). A 100 × 100 × 100 grid representing a maximum of 10^6^ cells was initialized with a single cell, a single blood supply for nutrients and a limited growth factor supply. Based on a literature survey and an informal sensitivity analysis, cells were able to acquire mutations at pre-defined probabilites. Acquired mutations included genetic instability (increased rate of subsequent mutagenesis), insensitivity to inhibitory signals, evasion of apoptosis, limitless replication, self-sufficiency in growth and sustained angiogenic signaling. Growth of the vascular system under angiogenic signaling was overlaid on the grid.

Simulated early onset tumors in this model were dominated by genetic instability while late onset tumors were driven largely by acquisition of limitless growth. The model further emphasized the importance to tumor growth of acquiring multiple different mutations and demonstrated large fluctuations in tumor heterogeneity and size as they developed. However, though it wasn’t required for initiation of tumor formation and development, angiogenesis was found to be the primary factor driving tumor growth beyond early stages. It is interesting that cells supporting angiogenesis did not necessarily dominate larger tumors. Instead other cell types with growth or survival advantages would “piggyback” their development off the increased blood supply stimulated by a nearby angiogenic sub-population (Spencer et al. 2006).

### Tumor growth

Pre-vascular tumor tissue growth can be modeled mathematically from macroscopic growth curve data. Tumor size often assumes a sigmoidal growth pattern which many earlier researchers interpreted as demonstrating the dominant role played by surrounding growth inhibitors (Cox et al. 1980). However, replacing inhibitors with an external supply of a hypothetical growth factor resulted in a more accurate simulation of tumor morphology in simple PDE models (Castro et al. 2005). More recent studies have added autocrine stimulatory factors such as EGF to the earlier models to more accurately reflect spatial characteristics of tumor tissue including the thickness of the proliferating rim in tumor spheroids (Bajzer and Vuk-Pavlovic, 2005).

Epidermal growth factor receptor (EFGR, also known as ErbB1) overexpression has been strongly implicated in highly malignant brain tumors. A multi-scale agent-based model has been developed by Deisboeck and colleagues over the past several years to investigate the relationship between EGFR dynamics, tumor cell proliferation and cell migration (Mansury et al. 2002; Mansury and Deisboeck, 2003; Athale et al. 2005). The model simulated intracellular molecular interactions through a system of ODEs in an explicitly-compartmentalized system representing individual cells and incorporating paracrine and autocrine TGFα signaling as well as nutrient supply. This led to a simple representation of the phenotypic decision of the cell; whether to rest, proliferate or migrate. Motility was then simulated by placing cells on a CA-type lattice overlaid with nutrient and signaling molecule concentrations.

The model confirmed previous experimental findings that increasing EGFR density on the cell surface correlates with an increase in the rate of tumor expansion (Berens et al. 1996). The model also suggested that the early switching of cells in such aggressive tumors from proliferative to migrating behavior may be the result of EGFR signaling and suggested that proteomics data should be added to transcriptional analysis in making predictive assessments of tumor dynamics (Athale and Deisboeck, 2006). A subsequent version of this model explicitly incorporated an updated molecular model of the cell cycle as well as the effects of hypoxia on the division rate and expanded the lattice into three dimensions, thereby necessitating the use of PDEs (Athale and Deisboeck, 2006). This version largely corroborated the earlier findings. It also led to the recommendation that the spatio-temporal dynamics of protein-gene interactions should be monitored diagnostically to distinguish between different molecular network states that nonetheless have highly similar cell phenotypes.

In epithelial tissue, normal growth is regulated by a complex interplay between inhibitory mechanisms and growth stimulating signals. Many tumors are initiated when cells make a transition from stable epithelial behaviour to expanding mesenchymal growth (Thiery, 2002). A lattice-free DCA biophysical model permitted the simulation of cell- and tissue-shape changes under pressures of adhesion and deformation from neighboring cells and underlying extra-cellular matrix (Galle et al. 2005). Displacement and deformation forces were modeled using Langevin equations incorporating both deterministic intercellular and stochastic forces with constants derived from the literature or directly from experiments. The model was validated using about 10^4^ cells in a full 3-D simulation, though most data was collected using a 3-D monolayer. Growth inhibitory cell-cell interactions were modeled as well as cell-substrate division inhibition and *anoikis*, a form of programmed cell death initiated when cells lose contact with their underlying matrix. The strength of the cell-substrate adhesion was found to be critical in inhibiting formation of spheroids atop the epithelial layers (Galle et al. 2005).

In a subsequent paper, the model predictions were compared to growth patterns of cultured tumor cells overexpressing alternative isoforms of the EGF receptor, CD97 (Galle et al. 2006). Over-expression of one particular CD97 isoform (EGF 1,2,5) stimulated single-cell extracellular matrix proteolysis and motility. However, it had no affect on cell-doubling times. Simulations confirmed these findings and added several other important observations: 1) directed migration away from the tumor center led to much more rapid invasion of surrounding tissue, 2) modifying the endogenous rate of cell cycling or induction of apoptosis from normal cells had little effect on tumor invasion (but, paradoxically, slowing the cell cyle sufficiently, permitted more of them to escape contact inhibition and enter a rapid growth phase), and 3) if the rate of migration increased as a result of reduced contact inhibition from neighboring tumor cells rather than as a result of growth induction from surrounding tissues the clonal population of the simulated tumor matched actual tumors more closely. This was confirmed by an experiment showing CD97 expression was lower in confluent (contact-inhibited) cells than in isolated cells in culture (Galle et al. 2006).

At the molecular scale, the dynamics of EGFR ligand binding and receptor dimerization is still largely unknown. A dynamic cellular automata (DCA) model incorporating Monte Carlo methods has been used to simulate EGF receptor activation and to compare the predictions to single-particle tracking studies (Mayawala et al. 2005). Receptors were randomly initiated into two-dimensional lattices of 100 × 100 or 250 × 250 cells (representing the plasma membrane) at high (5,500/μm^2^) or low (125/μm^2^) densities. Diffusion probabilities were calculated using random walk theory from diffusivity constants while reaction probabilities were similarly calculated from kinetic constants. To improve computational efficiency only lattice-sites containing receptors were randomly selected for movement or reaction at each time step. Three possible pathways to EGFR activation were simulated: 1) dimerization followed by ligand-binding, 2) ligand-binding followed by heterodimer formation followed by more ligand-binding, and 3) ligand-binding followed by homodimer formation of ligand-receptor complexes. The model demonstrated a dependence on both ligand and receptor concentration as to the specific mechanism that was most favored.

Tumors do not always develop in the relatively uniform mesenchymal environment. Lung adenocarcinomas originate and develop within the epithelia of the alveoli and the resulting tumor has a distinct non-spherical shape (Kerr, 2001). The alveolar epithelia may be modeled as an extended series of tubular sacs and a simplified simulation of tumorous growth along a single sac has been implemented recently in a system of PDEs (Marciniak-Czochra and Kimmel, 2007). In this model, cell proliferation was enhanced by a presumed growth factor which was produced by the surround and bound at the cell surface. The growth factor then spread by an unidentified inter-cellular diffusion process to adjacent cells. The growth factor was not consumed by cells but was supplied continuously. Under these conditions, cells exhibited a long latency period but eventually entered a phase of exponential increase in some regions with concomitant exponential decrease in others, eventually leading to a “chaotic” growth profile (Marciniak-Czochra and Kimmel, 2007).

Whether in a mesenchymal or epithelial environment, tumor growth is actively inhibited by interactions with NK cells and cytotoxic T cells of the immune system. A hybrid CA-PDE model has recently been used to simulate the complex response of tumors to growth signals while under attack by the immune system (Mallet and De Pillis, 2006). Cells were represented on a square grid supplied by nutrients along the top and bottom edges. Each grid square contained only one cell, either a cancer cell, an NK cell, a cytotoxic T cell or a normal cell. PDE equations governed the diffusion of two small-molecule nutrients; one required for cancer cell survival, the other for division. At each time cycle, diffusion of the nutrients was first simulated and the new concentrations imposed on the grid. Then cells responded to both the nutrient level as well as to neighboring cells according to specified CA rules. The authors found that, while basic tumor growth was accurately modeled, the tumor and immune cell populations were unexpectedly sensitive to different recruitment and killing parameters for cytotoxic T cells. Depending on the exact values, both tumor and immune cell populations could oscillate wildly in the model, accurately portraying oscillations found experimentally in diseases such as non-Hodgkins lymphoma (Mallet and De Pillis, 2006).

### Avoiding apoptosis

Escaping apoptotic signaling is an important mechanism cancer cells use to avoid being cleared from the body early in tumor develpoment (Hanahan and Weinberg, 2000; Abbott et al. 2006). In normal cells the apoptotic response can be induced by ligand binding to the membrane-bound FAS/CD95 death receptor which induces formation of the death-inducing signaling complex (DISC) and results in the production of activated forms of caspase 8 and caspase 3, the major apoptotic effectors. A critical survey of the literature and available databases led to the development of a complex ODE model of the CD95-inducible apoptotic pathway which included about 70 molecules, 80 equations and 120 unknown parameters (Bentele et al. 2004). Mitochondria and protein degradation components were subsequently packaged into “black-box” components, defined by their input-output behavior, in order to make a tractable model. A subsequent sensitivity analysis reduced the number of unknown global parameters from 58 to 18.

Application of the model led to the discovery of a threshold mechanism in apoptotic signaling. The c-FLIP protein inhibits DISC activity by strongly binding to the caspase-8 activating site of DISCs. Below threshold levels of CD95 signaling, c-FLIP binding was found to limit the number of active DISCs. As a result no cells died. Above the threshold level, the number of activated DISCs exceeded the cytoplasmic c-FLIP pool and apoptosis was predicted to occur in all cells. Following chemotherapeutic treatment, many cancers develop apoptotic resistance, which may be due to the abnormally high c-FLIP expression found in certain cancer cells (Micheau, 2003). Subsequent experiments have shown that overexpression of a transcription factor (E2F1) down-regulated c-FLIP in cultured human lung adenocarcinoma cells and sensitized them to FAS-induced apoptosis and T lymphocyte attack (Salon et al. 2005).

NF-κB (Nuclear factor—kappa B) is an important downstream transcription factor also implicated in the regulation of apoptosis as well as in cell signaling, growth and response to stress. It has been identified as a therapeutic target in chronic inflammatory diseases as well as in cancer (Yamamoto and Gaynor, 2001). An ODE model of the regulatory factors of NF-κB has been developed incorporating NF-κB, three IκB inhibitory isoforms (IκBα, -β and -ɛ) and the activating IκB kinase, IKK (Hoffmann et al. 2002). The resulting model consisted of 24 ODEs. It included rates of formation, degradation and transport of all biologically-relevant monomers, as well as dimeric or trimeric complexes in explicit nuclear and cytoplasmic compartments. Parameters were derived from the literature, previous experiments or by fitting to a knock-out cell line using a genetic search algorithm. Finally, parameters were adjusted to fit wild-type cell data.

Simulations using the adjusted parameters led to the surprising discovery that the different inhibitory proteins (IκBα, -β and -ɛ) act together to produce different components of NF-κB expression levels. By itself, IκBα results in strongly oscillatory levels of NF-κB expression, while increasing levels of IκBβ and IκBɛ led to first a dampening of the oscillations and then a levelling out of NF-κB expression at a plateau. This mechanism predicted that transient stimulation by TNF-α should lead to prolonged expression of NF-κB resulting in sustained production of downstream genes and protection from apoptosis.

### Angiogenesis

In general, the growth of solid tumors is limited to about 0.5 mm diameter without access to an oxygenated blood supply. To develop beyond this size, tumors may stimulate the growth of new blood vessels. This process is called angiogenesis. It is dependent upon the secreted growth factor Angiopoietin 2 (Ang2) binding to the transmembrane Tie2 receptor tyrosine kinase (Bach et al. 2007). In the presence of vascular endothelial growth factor (VEGF), along with other proliferative and migratory signals, this causes sprouting of new vasculature from existing vessels.

An ODE simulation of the effect of this vascular remodeling on tumor growth has recently been developed (Arakelyan et al. 2002). The model incorporated starvation-induced VEGF expression in tumors as the sole angiogenic factor but also accomodated the destabilization of mature vessels and regression of immature vessels by Ang2. While formation of new immature vasculature led to a strictly monotonic exponential growth of tumor tissue, incorporating vessel regression and vascular maturation into the model led to oscillations in both vessel and tumor tissue volumes. This led the authors to suggest that treatment with both anti-angiogenic and anti-maturation drugs might be more efficacious than mono-therapeutic approaches. This theoretical result was found to be consistent with phase 3 clinical data for the anti-angiogenic drug Avastin (Garber, 2002).

Angiogenic factors are secreted by hypoxic cells on the periphery of the central necrotic region of the tumor (Bach et al. 2007). The cellular response to hypoxia is largely regulated by the α subunit of HIF1, the heterodimeric hypoxia-inducible transcription factor. High nuclear levels of HIF1α have been associated with higher grade gliomas independent of hypoxic induction (Zagzag et al. 2000). Under normal oxygenation levels, HIF1α remains in the cytosol where it is first prolyl-hydroxylated by prolyl hydroxylase domain 2 enzyme (PHD2), then ubiquitylated and subsequently degraded by the proteasome. As a result cytosoloic HIF1α has a very short estimated half-life (five to eight minutes). In hypoxic conditions the cytosolic HIF1α escapes hydroxylation and enters the nucleus, where it binds with HIF1β/ARNT and activates the angiogenic pathway, including VEGF and its receptor VEGFR2/Flk1. Hydroxylation of HIF1α by PHD2 in the nucleus blocks binding to HIF1β/ARNT and reduces transcriptional activation by HIF1α.

The hypoxic response pathway was modeled by a system of ODEs reflecting the molecular kinetics of 17 compounds and validated to data from several independent experiments (Qutub and Popel, 2006). The model demonstrated both a rapid, switch-like response to low oxygen and a slower, more gradual one, depending on the presence of cytosolic iron, ascorbate and PHD2. Based on these findings iron supplementation, ascorbate supplementation, or a combination of both were compared to PHD2 targeting as alternative therapeutic approaches to increasing HIF1α hydroxylation and reducing its transcriptional activation under hypoxic conditions. Ascorbate supplementation alone was found to be very effective at increasing hydoxylation levels of HIF1α, but the effect was considerably reduced (from 60% to only 3%) when iron supplementation was also available (Qutub and Popel, 2006). Iron supplementation and increased expression of PHD2 were equally effective at increasing HIF1α hydroxylation. These findings might suggest that iron supplementation coupled with increased ascorbate should be a cost-effective therapeutic approach to inhibiting HIF1α angiogenesis of hypoxic tumors. However the authors were quick to point out that their model has several significant limitations, including unknown kinetic reaction rates, unknown effects of the acidic tumor microenvironment, different HIF1α binding affinities for iron versus oxygen and other, recently-characterized proteins that effect the rate of hydroxylation of HIF1α.

### Therapeutics

Therapeutic approaches to cancers include surgical removal (in whole or part), partial or whole body irradiation and a vast array of chemotherapies. Chemotherapeutic agents may be broadly classified as cell-cycle phase-specific, cell-cycle non-specific, or cytostatic/anti-angiogenic (Gardner, 2002). Phase-specific drugs include methotrexate and 5-fluorouracil, both of which act by blocking DNA synthesis during S-phase in rapidly dividing cancer cells. Cylcophosphamide and doxorubicin are examples of cell-cycle non-specific drugs; these drugs interfere with DNA function by covalent modification or non-covalent intercalation. Cytostatic drugs include tamoxifen and herceptin, which act as antagonists to specific growth factor receptors whose activity is necessary for tumor development. Avastin and sorafenib are examples of anti-angiogenic drugs which work by inhibiting VEGF activity either by direct binding or by blocking downstream signaling.

Chemotherapeutics are frequently administered in combination, because tumors are comprised of heterogeneous cell populations with different metabolic profiles and susceptibilities to attack by drugs. Gardner developed a kinetically tailored treatment (KITT) model which utilized a pair of ODEs (plus other supporting equations) to incorporate rates of tumor genesis, growth, apoptosis, necrosis, drug-induced death, development of drug resistance, cytotoxic side effects and drug pharmacokinetics (Gardner, 2002). Tumor growth was modeled either as exponential or Gompertzian and a variety of treatment regimens were tested, including all possible combinations of six relevant drugs under either standard or alternative schedules. Nearly 27,000 tumors were modeled over a wide range of growth and survival parameters. Including cytostatic drug administration with cytotoxic drugs substantially increased the effectiveness of the treatment whether the cytostatic component was administered under the standard or alternative schedule. In addition, rapidly dividing tumors responded optimally to treatment with two different cell-cycle phase-specific drugs while slower developing tumors responded better to treatment with two cell-cycle non-specific drugs.

The effectiveness of this two-component chemotherapeutic approach has been verified by a simple delayed differential equations model, relating tumor growth, immune system attack and drug-induced cell cycle inhibition (Villasana and Radunskaya, 2003). In this model, cells were subdivided into tumor interphase or mitotic subpopulations and immune system components. Treatment with a cell-cycle specific inhibitor arrested cells in mitosis where they could die due to either a failure to complete the cycle or to cytotoxic immune system effects. This model demonstrated that delaying cells in mitosis may actually lead to instability in cell populations if the delay does not lead to rapid tumor cell death. This confirmed the previous suggestion that combining cytostatic and cytotoxic drugs might lead to a more effective therapeutic approach than with either seperately (Gardner, 2002).

Similar findings were reported by Crispini and colleagues in their model of the interaction between tumor morphology and invasiveness and the micro-vasculature that develops under angiogenic conditions (Cristini et al. 2005). They used a hybrid system of 2-D PDEs to model tumor growth, nutrient/oxygen diffusion and tissue pressure, while the vasculature was represented by a discrete DCA superimposed on the grid (Zheng et al. 2005). In this model hypoxia and extracellular acidosis associated with poor vascularization of the central region of the tumor led to reduced cellular adhesion. Coupled with natural variations in vascular density, this caused non-uniform cell proliferation and migration leading to a “diffusional instability” that resulted in a highly-invasive tumor morphology. Neo-vascular suppression merely exacerbated this condition while “vascular normalization” led to improved nutrient and oxygen delivery to the tumor, resulting in stability of the spherical morphology and reduced invasiveness (Cristini et al. 2005).

However, anti-angiogenic therapies, rather than suppressing all vascular extension into the tumor, have actually been shown to normalize tumor vasculature by pruning immature vessels (Tong et al. 2004). Recent analysis has shown that this vascular pruning early in anti-angiogenic treatment is responsible for reducing interstitial fluid pressure and improving convection within the tumor while reducing convection from the tumor into its periphery (Jain et al. 2007). These improvements in convection within the tumor also permit improved delivery of co-administered cytoxic drugs to the tumor cells, leading to improved targeted killing of the cancer and confirming the benefits of two-component therapies (Tong et al. 2004).

In addition to optimizing therapeutic components and dosages, another challenge facing clinicians relates to selecting the most efficacious administration schedule. For example, in order to effectively prevent mammary carcinogenesis in HER-2/neu transgenic mice the Triplex vaccine must be administered beginning at six weeks after birth and repeated every two weeks for the entire lifespan of the mouse (Lollini et al. 2006). An ABM simulation was used as an evaluation model, driven by a genetic algorithm to test whether this arduous vaccination protocol could be optimized (Pappalardo et al. 2006). While “best guess” approaches resulted in 27% fewer repeat vaccinations, the genetic search algorithm discovered a protocol which reduced the number of vaccinations by 44% over the original. Experimental verification of this suggested protocol is underway.

Novel cancer therapeutics can have even more complex pharmacodynamics. For example, radio-virotherapy combines radiation therapy with the increased sensitivity of cancer cells to some viral infections. A viral genome (e.g. measles) is altered so that infection of a cell also introduces the gene encoding a transmembrane ion import pump. Expression of the ion pump in infected (but not dead) cells causes them to concentrate intravenously admitted radioactive ions leading to tumor regression (Dingli et al. 2004). The interaction of tumor cells, replicating virus, radioactive isotope and the immune system has recently been modeled in a complex compartmentalized system of ODEs to better understand the interaction between these components so that therapeutic outcomes may be optimized (Dingli et al. 2006). The system was partially discretized so that any population of cells or viruses below one was automatically set to zero; this allowed for the complete elimination of tumors and viruses in some simulations. Following validation of the model and estimation of parameters lacking experimental values, simulations led to the following conclusions: a) radiotherapy should begin within seven days of viral administration, b) initial viral dose partially determines response time to the therapy but more than five-fold increases over initial levels were not beneficial, and c) the optimal dose for radioactive iodide was within a narrow window (plus or minus 20%) and halving the dose did not result in complete loss of the tumor. In the absence of effective modeling, arriving at these same conclusions would have involved years of clinical trials.

## Conclusion

Although a number of important insights have already resulted from the relatively simple and non-comprehensive models presented above, it is clear that a number of challenges still exist. One of the most obvious is the lack of precise, quantitative data for gene and protein networks so that realistic parameters can be entered into these models. While a considerable amount of transcriptional and translational data is available, good quality metabolic data lags far behind. The situation is complicated in metazoans because, even in a disease state, not all cells in a tissue will express the same genes, proteins or metabolites. Single-cell isolation and analysis, including lab-on-a-chip and protein-nanoarrays, may improve this situation somewhat.

Ultimately, models of living systems attempt to simulate extremely complex mixtures of proteins, genes and metabolites. Approximations to the actual reactions occuring in such a complex mixture are made of necessity and the resulting simplifications may lose important, relevant information. For example, when modeling new candidate drugs, it may be impossible to predict all potential undesirable interactions with non-target molecules. While network models may be able to help identify some of these interactions based on resulting physiological changes, only accurate prediction of all possible aberrant reactions at the molecular level could obviate this issue entirely.

The proliferation of approaches and implementations makes it difficult to select the “best” approach for any particular modeling problem (see Fig. 1). Nevertheless comparisons between different formalisms for specific purposes have been made (Stromback and Lambrix, 2005). Several groups are actively collaborating (or, sometimes, competing) to develop a consensus approach to representing biological pathways and biochemical systems. A Unified Modeling Language (UML) to easily enable the hierarchical construction of hybrid models with explicit modeling of stochastic processes, compartmentalization and diffusion has been proposed (Webb and White, 2005). This formalism borrows heavily from the object-oriented experience in developing reusable, hierarchical program modules and is similar to a complex hierarchical, agent-based model.

Many researchers support the move towards a clear, consistent formalism to depict biological processes. Kitano has defined a System Biology Graphical Notation (Kitano et al. 2005) which has been encapsulated in the SBML-compatible program CellDesigner and used to successfully model the complex EGFR pathway (Oda et al. 2005). Other approaches to supporting hybrid combinations of different formalisms have been described above. As groups supporting one particular formalism over another point out the advantages of their approach, their best suggestions are often readily incorporated into the most prevalent formalisms. To some extent, there is a great deal of functional convergence among the different approaches and the best selection may be whichever one is “most natural” to the modeler without losing any of the desired power.

In recognition of the deleterious effect this proliferation of approaches has on the advancement of modeling, particularly in relation to cancer research, the National Cancer Institute has recently established the Integrative Cancer Biology Program. The Center for the Development of a Virtual Tumor (CViT) at the Massachusetts General Hospital in Boston is one member of this program (Deisboeck et al. 2007). The CViT is attempting to establish a community of investigators who will work towards the development of a multi-scale simulation platform and repository in support of modeling cancers. In addition to the model repository, CViT will provide tools that support world-wide collaborative efforts in modeling cancer and that provide a framework for semantic descriptions of models independent of their implementation. Effort, such as this, will be crucial in order for computational systems biology researchers to benefit from, and build upon, each other’s experiences.

## Figures and Tables

**Table 1:** Partial list of computational systems biology simulation software packages.

Method	Package	URL and reference
ODE, PDE, SSF	Cell Designer	http://www.celldesigner.org/index.html (Kitano et al. 2005)
	CellWare	www.cellware.org (Dhar et al. 2004)
	Dynetica	http://www.duke.edu/~you/Dynetica_page.htm (You et al. 2003)
	E-Cell	http://www.e-cell.org/ (Tomita et al. 1999)
	Gepasi	http://www.gepasi.org/ (Mendes, 1993)
	SmartCell	http://smartcell.embl.de/ (Ander et al. 2004)
	VCell	http://www.vcell.org (Loew and Schaff, 2001)
	MesoRD	http://mesord.sourceforge.net/index.phtml (Hattne et al. 2005)
	Dizzy	http://magnet.systemsbiology.net/software/Dizzy/ (Ramsey et al. 2005)
Petri Net,	Snoopy	http://www-dssz.informatik.tu-cottbus.de/index.html?/software/snoopy.html
	CPN Tools	http://wiki.daimi.au.dk/cpntools/cpntools.wiki (Lee et al. 2006)
	Cell Illustrator-Animator	http://www.gene-networks.com (Peleg et al. 2005)
DCA and ABM	CancerSim	http://www.cs.unm.edu/~forrest/software/cancersim/ (Abbott et al. 2006)
	MCell	http://www.mcell.cnl.salk.edu/(Stiles and Bartol, 2001)
	SimCell	http://wishart.biology.ualberta.ca/SimCell/ (Wishart et al. 2005)
	AgentCell	http://flash.uchicago.edu/~emonet/biology/agentcell/ (Emonet et al. 2005)
Hybrid	Cell++	http://www.compsysbio.org/CellSim/ (Sanford et al. 2006)
	CellML	http://www.cellml.org/ (Bhalla and Ravi Iyengar, 1999)
